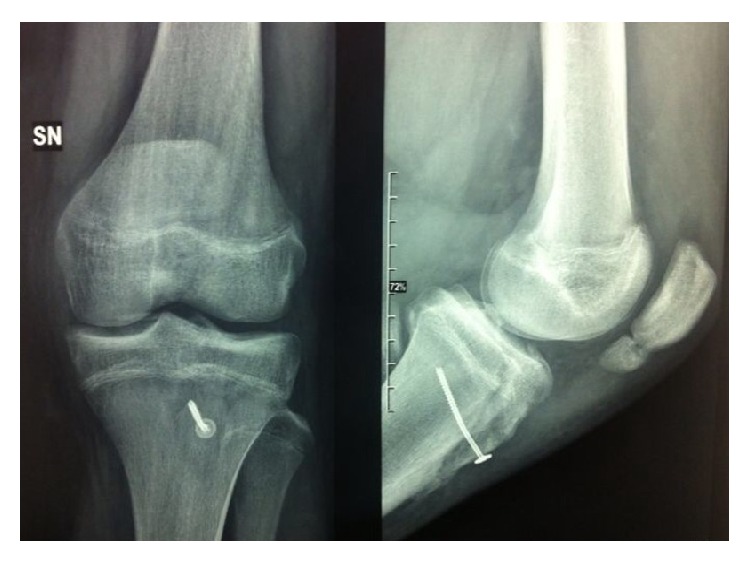# Tibial Tubercle Fracture in a 14-Year-Old Athlete with Bilateral Lower Pole Bipartite Patella and Osgood-Schlatter Disease

**DOI:** 10.1155/2015/815061

**Published:** 2015-02-16

**Authors:** Fabio Pascarella, Antonio Ziranu, Giulio Maccauro

**Affiliations:** Department of Orthopaedics and Traumatology, Catholic University of Sacred Heart, 00168 Rome, Italy

## Abstract

We present a case of tibial tubercle fracture in a young male athlete with both bilateral bipartite patella at the lower pole (Saupe type I) and Osgood-Schlatter disease. Open reduction and internal fixation were performed to restore the extensor mechanism of the knee.

## 1. Introduction

Knee injuries in young athletes include not only the typical adult bone injuries, ligament injuries, and cartilage injuries, but also the growth plate lesions.

Several reports of multiple osteochondroses are found in scientific literature.

We treated a patient with multiple osteochondroses: bipartite patella and Osgood-Schlatter disease in the left knee and bipartite patella in the right knee.

Avulsion fractures of the tibial tubercle are rare injuries in the pediatric population with a reported incidence between 0.4% and 2.7%; they represent less than 1% of all physeal injuries and are usually seen in adolescent males approaching skeletal maturity with well-developed quadriceps musculature [1].

The mechanism leading to tubercle avulsion is mainly represented by aggressive knee flexion during quadriceps contraction or aggressive quadriceps contraction when the ipsilateral foot is fixed [2].

Ogden classified tibial tuberosity fracture modifying the more commonly used Watson-Jones classification [3, 4].

Bipartite patella is a well-known osseous variant. Saupe in 1943 described three types of bipartite patella: type I at the inferior pole (5%), type II at the lateral margin (20%), and the most common type III, at the latero-superior pole (75%) [5].

Type I is rare and its existence as a pathological entity separate from fragmentation due to Sinding-Larson-Johansson disease or stress fracture has been called into question by Oohashi et al. in a large series of bipartite and multipartite patellae [6].

We report a case of multiple osteochondroses complicated by tibial tubercle fracture on the left knee.

To our knowledge, this is the first reported case of tibial tubercle fracture in a patient with bipartite patella.

## 2. Presentation

A 14-year-old male soccer player was admitted to the “First Aid Department” with left knee injury, showing anterior pain and swelling. During a soccer game he suddenly developed acute pain at the left knee immediately after kicking with his right foot. There was no direct trauma, but quadriceps contraction while the ipsilateral foot was fixed.

The patient practiced sport activities 4 days a week. He denied any history of pain or swelling at the left knee during the previous year in which he had a growth spurt of 12 cm.

## 3. Examination

Physical examination showed swelling of both the left knee and the calf, together with pain at the tibial tubercle. The left patella was higher than the contralateral side. The patient was unable to contract left quadriceps, extend his left knee, or walk. On the right knee, there was no swelling, pain, or regular quadriceps contraction: the right knee had a complete ROM. The patient's weight was 85 kg and his height was 180 cm (BMI 26).

## 4. Imaging

**Figure 1:**
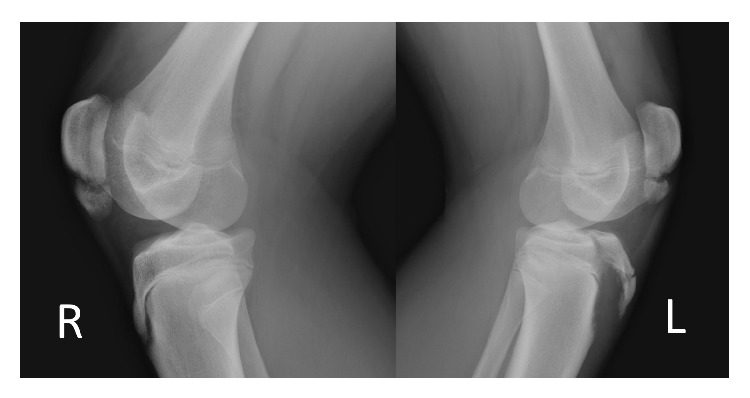


**Figure 2:**
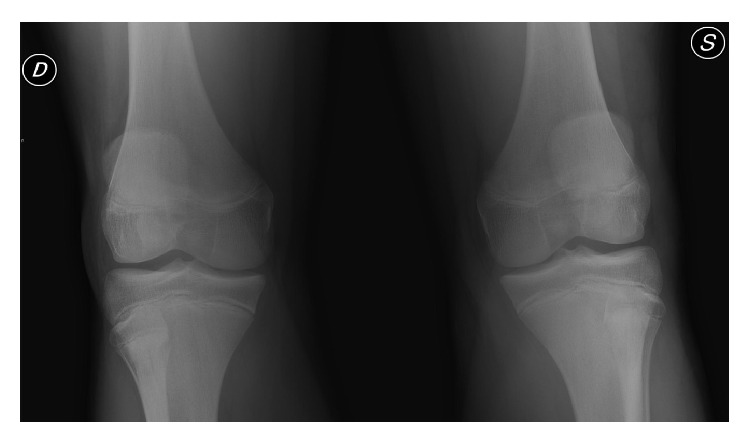


Radiographs showed patella alta, tibial tubercle fracture (Ogden III A), radiological findings for Osgood-Schlatter disease (the tubercle was elongated and fragmented), and bipartite patella at the lower pole (Saupe type I) on the left knee and bipartite patella (Saupe type I) on the right knee (Figures 1 and 2).

## 5. Treatment

Operative treatment was indicated, and approximately 6 hours after trauma the tibial tubercle avulsion (Ogden III A) was fixed with a screw after reduction and removal of periosteum from the fracture bed.

Second generation cephalosporin was administered 30 minutes before surgery.

Postoperatively the left knee was immobilized in a plaster and low weight heparin was administered for 30 days.

X-ray was performed 15 days and 3 months after the operation.

The patient, 2 months after surgery, started sport activities without medical advices and, at the scheduled follow-up at 3 months, he had no pain and had good quadriceps contraction and complete ROM. His left knee was not swelling and the X-ray showed radiographic union (Figure 3).

At 6 months after surgery, the screw was removed. The screw was bent by 15° but was not broken.

## 6. Discussion

Tibial tuberosity develops from a secondary ossification center. In contrast to the proximal tibial epiphysis, which develops in compression, the tibial tuberosity develops in traction [7].

The proximal tibial physis has been shown to close in a posterior to anterior direction with subsequent progression of closure toward the tubercle epiphysis which is closing in a proximal to distal direction as well [7, 8].

Tibial tuberosity fracture is more frequent in males and on the left side [9].

A definitive correlation between Osgood-Schlatter disease and tibial tuberosity fracture has not been shown. However, Osgood-Schlatter disease has been reported as an associated finding with tibial tubercle fracture in nearly every study [7].

The patella initially ossifies between 3 and 5 years of age. Ossification starts from multiple foci which rapidly coalesce [8].

Secondary ossification centers appear as bi- or tripartite patella with fibrocartilaginous continuity despite of osseous discontinuity. Late state of maturation is reached at the age of 10–12 years.

Oohashi et al. in their study reported that bipartite patella was more common in males (77%) than in females (23%) and that bilateral involvement occurred in 25%. Unilateral involvement was almost the same in both knees [6].

Painful bipartite patella is occasionally observed in adolescents and young adults [10].

Until now, only three authors reported Saupe type I bipartite patella in literature but only in patients younger than 12 [11–13].

Tauber et al. say that traumatic separation of bipartite patella occurs when the cartilaginous connection between the ossification centers is weakened by recurrent microtrauma, inflammation, or chronic stress [14].

Our patient showed multiple osteochondroses, and these disorders in literature are related to excessive and repeated stress in the extensor mechanism in growing athletes. The pathogenesis of these diseases is due to the traumatically induced disruption that occurs more easily when rapid growth spurt is present.

Our patient was 14 years old and it is known that patella maturation is reached at the age of 10–12 years, while the physiodesis occurs at a later age (approximately 14 years old).

His growth spurt and sport activities caused a chronic stress on the extensor mechanism.

He reported a tibial tubercle fracture after aggressive quadriceps contraction with the ipsilateral foot fixed to the ground: the traction forces acted on all the extensor mechanism, but the cartilaginous connection between the ossification centers of the patella was more resistant than the physeal/metaphyseal interface.

In our opinion, this led to a tibial tubercle fracture instead of patella traumatic separation.

## Figures and Tables

**Figure 3:**